# The Bristol Eye Hospital—An Historical Perspective

**Published:** 1986-12

**Authors:** V. J. Marmion

**Affiliations:** Consultant Ophthalmologist, Bristol Eye Hospital


### The Bristol Eye Hospital – an historical perspective

**V. J. Marmion**
Consultant Ophthalmologist, Bristol Eye Hospital.

The completion of the rebuilding of the Bristol Eye Hospital coincided with the 175th anniversary of its foundation in Lower Maudlin Street at the premises of the Blind Asylum. The new building marks an important step forward in the standard and quality of ophthalmic care within the City, to the wider boundaries of the Avon area, and as a referral centre in the South West.

Its foundation, in 1810 was, in a major part, due to the endeavour of Dr William Goldwyre, a Surgeon Occulist in the City who had had his training initially in Bristol, then in London. He later travelled extensively on the Continent to expand his experience in the chosen specialty. In recognition of the valuable service he contributed to the City he was elected a Freeman of the City in 1816.

At its foundation in 1810, the Eye Hospital served an important need in relation to several communicable diseases so prevalent amongst the poor, notably the infectious disease of Egyptian Ophthalmia (Trachoma) and Smallpox. Considerable anxiety was expressed in the early years of the hospital about the incidence of Smallpox and the failure of the poor in the City to have adequate vaccination in spite of its proven efficacy. In the first half of the nineteenth century smallpox was a major cause of blindness. Some two thousand patients a year attended the Outpatient Department and a few were admitted for inpatient care. Surgical treatment for conditions such as Cataract, Pterygium and Strabismus was undertaken and good results were reported.

The increase in the population of the City in the mid-nineteenth century coincided with the expansion of medicine and the specialty of ophthalmology in particular. A programme to extend the hospital commenced in 1884, this and its implementation over the next twenty years were primarily due to the efforts of Mr Francis Richardson Cross. The first requirement then as ninety years later, was an improved operating theatre. General anaesthesia had been regular use at the Eye Hospital since 1873 and the use of topical cocaine for local anaesthesia had been introduced in 1880. Diagnostic procedures were being rapidly expanded. The hospital was fortunate to have a Committee with considerable foresight implementing facilities such as gas light, electricity and a telephone at an early stage of their development. The improvements of 1884 to 1889 led to the establishment of a specific nursing facility and resident House Officers at the hospital. Mr Cross served a period as Dean of the Medical School during his early years in Bristol and it is not surprising that a University Department of Ophthalmology was established in 1900. In 1903 Mr Cyril Walker was appointed Lecturer.

By 1923 it was apparent that a new building was required. The planning, acquisition of land and the raising of funds meant that this was not completed until 1935. With the rapid change in the scope of ophthalmic care between 1950 and 1960, this building became obsolescent and led to the planning of the present building. The modern Bristol Eye Hospital reflects and incorporates the changing needs and developments of our time. The philosophy of the building is towards providing a high standard of care which is implemented as rapidly as possible thus reducing the time and number of visits a new patient has to make to the hospital. The Victorian Out-patient Department has given way to individual diagnostic rooms which are fitted with an abundance of instruments of high quality. All the important ophthalmic investigations are housed on site. The Casualty Department has ample space and the introduction of the Triage System, for grading patients, has improved the working of the Casualty and enlisted the understanding and co-operation of the patients.

Teaching facilities are provided in the Out-patient Department both in the individual rooms and in a special teaching room which is fully equipped. There are similar rooms on each of the In-patient floors. There is now a University Department housed in the hospital itself and this contains laboratory space and a seminar room. The University Department has a commitment towards the investigation of viral corneal disease, its immunological ramifications and corneal grafting.

Twenty five years ago some three thousand operations a year were undertaken in one small theatre. Today, a similar number of operations are being performed in three modern theatres fully equipped for microscopic surgery and with excellent facilities for general anaesthesia. There is now an excellent five bay recovery ward. A most welcome feature of the new theatre suite lies with the theatre sterile supply unit which caters for the whole hospital and has special facilities for the cleaning and maintenance of the fine ophthalmic instruments.

In the past fifty years, the Eye Hospital has been pleased to celebrate its important anniversaries with symposia which provide an opportunity to demonstrate the facilities available, the range of teaching undertaken and the research interests which pervade the institution. The Bristol Medico Chirurgical Society celebrated the opening of the Eye Hospital in 1936 and at that time was brought up-to-date on the new developments in relation to corneal grafting. The Surgeons at the Eye Hospital welcome the opportunity to meet with the Bristol Medico Chirurgical Society again and confidently expect that the new facilities, much appreciated by the patients and staff, will be of considerable interest to the Society as will be the active research programmes which are being undertaken.

